# Supplementary material for: A critical period of neuronal activity results in aberrant neurogenesis rewiring hippocampal circuitry in a mouse model of epilepsy
Source: Nat Commun. 2021 Mar 3;12:1423. doi: 10.1038/s41467-021-21649-8 (PMC7930276; doi:10.1038/s41467-021-21649-8)
Supplement: Supplementary file 1 — Supplementary Information [file 41467_2021_21649_MOESM1_ESM.pdf]

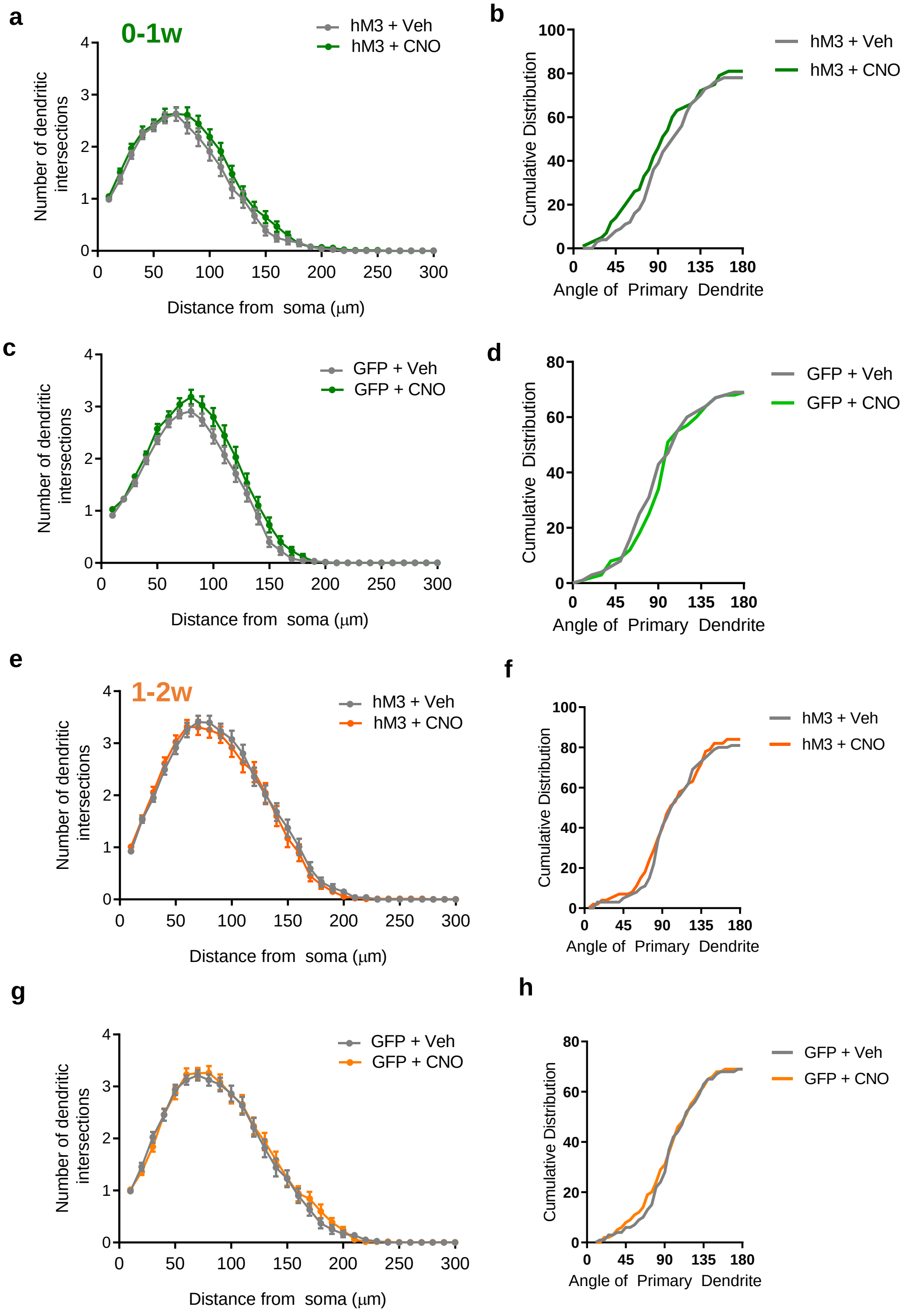

Figure S1. DREADD activation of new neurons does not alter dendrite morphology. For mice injected with hM3Dq or GFP at 0-1w or 1-2w dendrite morphology was measured. There was no difference in dendrite complexity in (A) hM3Dq mice given CNO or Veh. (B) Cumulative distribution for angle of primary dendrite in hM3Dq 0-1w group. (C) Sholl analysis and (D) cumulative distribution for GFP control group. (E-H) There was no change in dendrite complexity or primary dendrite angle of the hM3Dq activated group at 1-2w. (E) Sholl analysis and (F) cumulative distribution for hM3Dq mice given CNO or Veh at 1-2w. (G) Sholl analysis and (H) cumulative distribution for GFP control mice in 1-2w groups. ANOVA analysis and K-S statistical tests were used for analysis. Error bars denote SEM; n=5 mice per group.

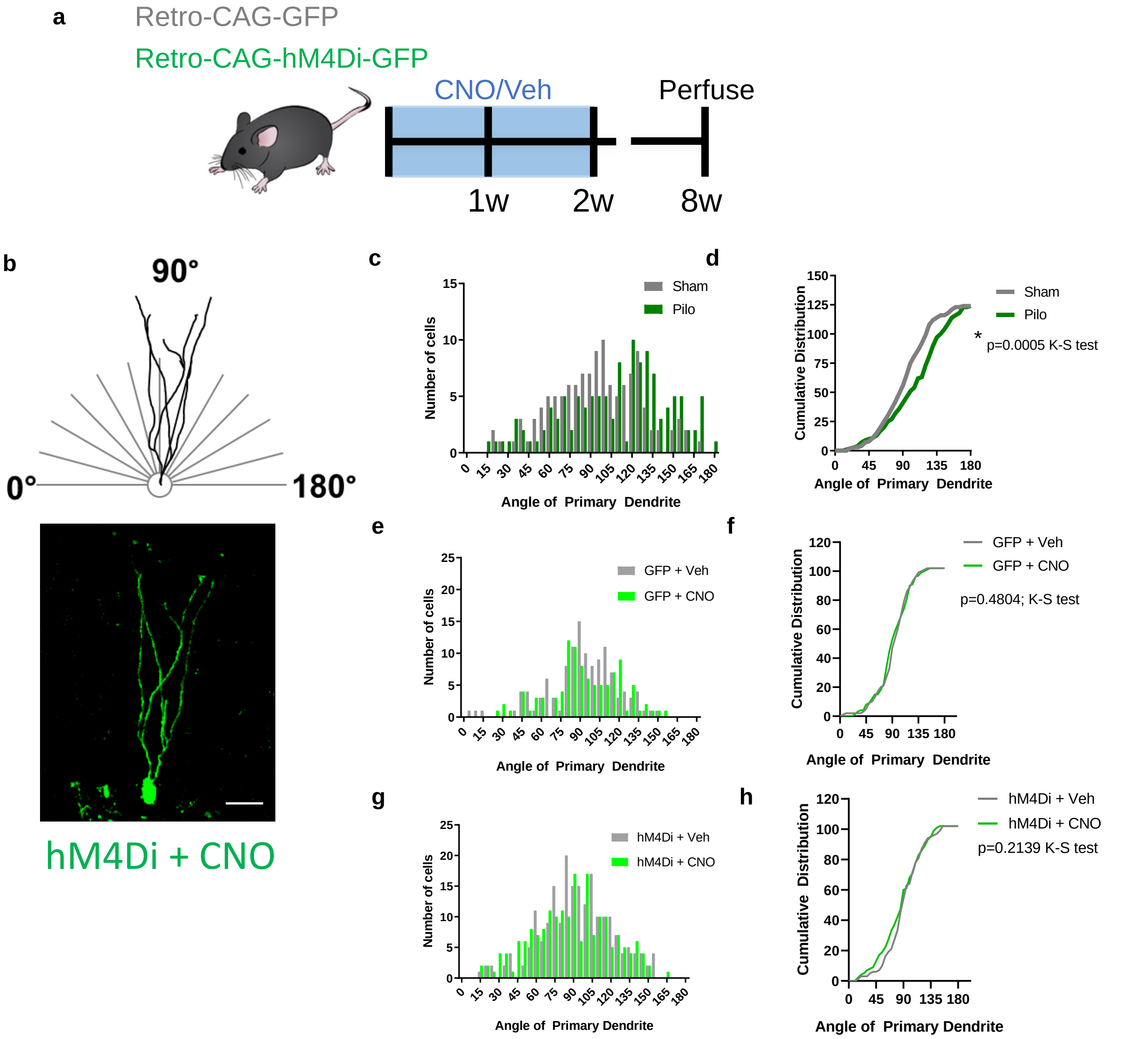

Figure S2. Silencing neurogenesis does not alter dendrite angle. (A). Mice were injected with retrovirus to express hM4Di in neural stem cells. CNO was administered daily for the first 2 weeks. (B) Representative trace and image. Scale bar represents 20µm. (C) Pilocarpine shifts the primary dendrite angle past 90 degrees. Histogram of dendrite angle. (Sham p=0.9027, K2= 0.2048) and (Pilo p=0.0640, K2=5.498) (D) Cumulative distribution of primary angle between sham and pilo groups. (E) There was no difference in dendrite angle in GFP. (GFP+Veh p=0.0037, K2=11.19 and GFP+CNO p=0.5183, K2=1.134). (F) Cumulative frequency of GFP group. (hM4Di+Veh p=0.0832, K2=4.972; hM4Di+CNO p=0.0957, K2=4.694(G) Distribution of angles from hM4Di. (H) Cumulative distribution of hM4Di group. *p<0.01, Kolmogorov–Smirnov test,. Test for normality uses the D'Agostio & Pearson test to report K2. n= 10 per group. All statistics calculated using two-tailed test.

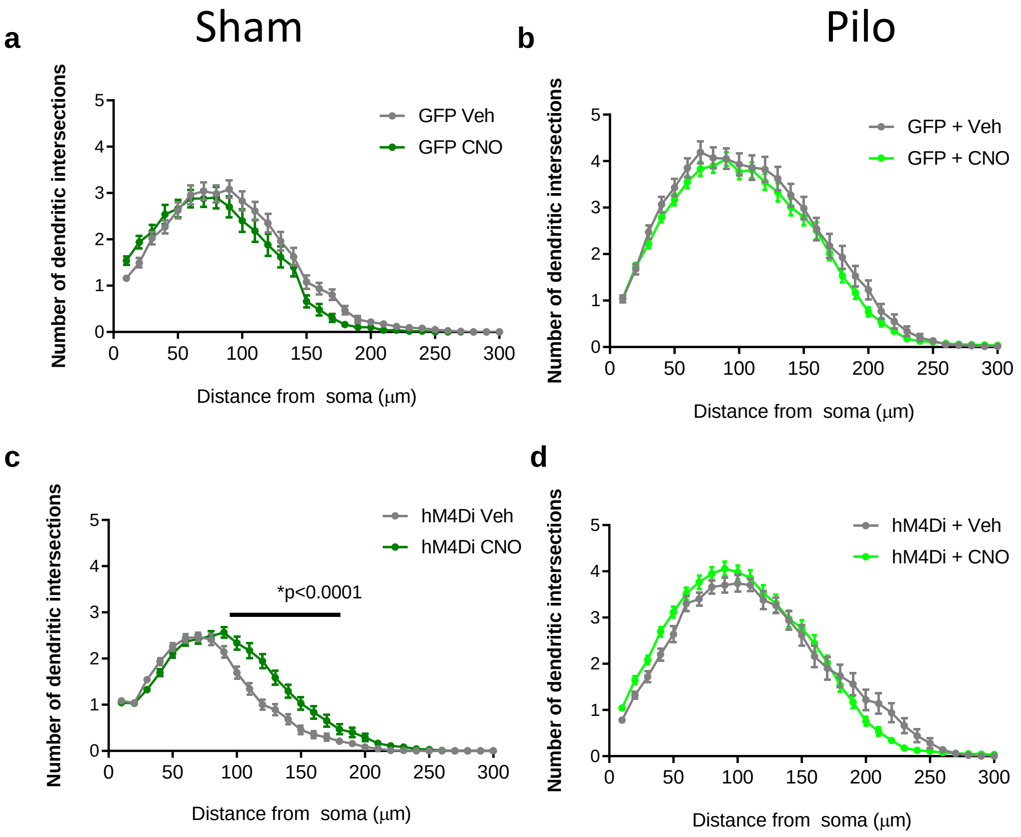

Figure S3. Silencing neurogenesis shifts dendrite branching in sham but not pilo. (A).Sholl analysis for sham mice in the GFP group. (B) CNO does not alter dendrite complexity in GFP group with pilocarpine. (C) Silencing neurogenesis shifts dendrite branching distally from the soma in sham group, (D) but that effect is lost in pilocarpine group. ANOVA with multiple comparisons. *p<0.05; n= 87(GFP+Veh;sham), 77(GFP+CNO;sham), 118(GFP+CNO;Pilo), 74(GFP+Veh;pilo), 50(hM4Di+Veh;sham), 55(hM4Di+CNO; sham), 50(hM4Di+Veh; pilo), 118(hM3Di+CNO;pilo). Error bars presented as SEM. All statistics calculated using two-tailed test.

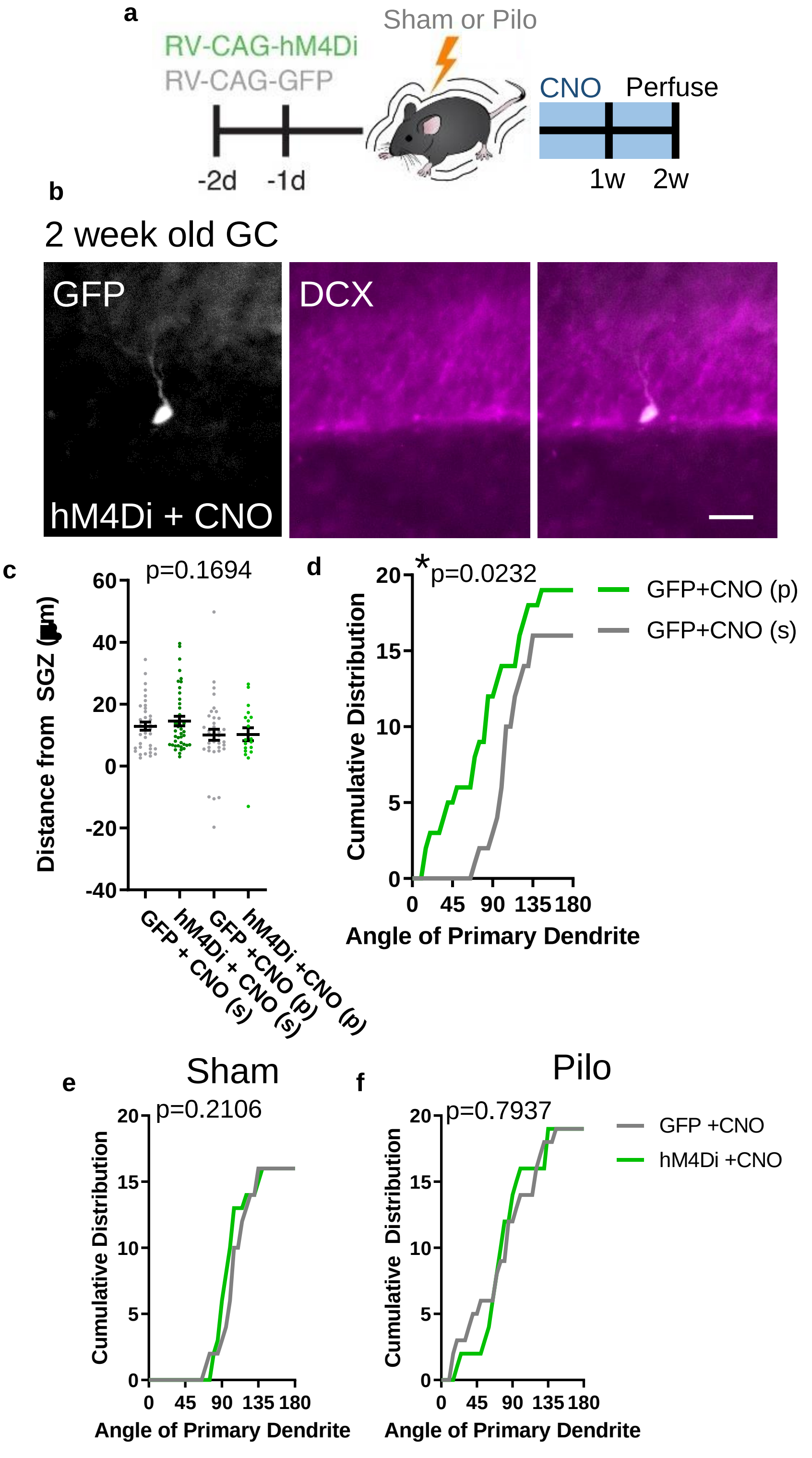

Figure S4. Morphology of 2 week old cells. (A) Experimental design (B) Representative image of 2 week old GC. Scale bar represents 20µm. (C) Quantification of migration distance. There is no significant differences in migration distance between sham (s) and pilo (p) groups. However, ectopic granule cells in the hilus are present at 2 weeks in the pilo group and there is a modest reduction in the hM4Di+CNO (p) group compared to the control GFP+CNO (p). Error bars represent SEM. *p<0.05, one-way ANOVA, (D) Cumulative distribution of primary dendrite. (E) At 2 weeks there appears a shift in primary dendrite angle, but hM4Di+CNO does not appear to change in either (E) sham or (F) pilo groups with CNO. *p<0.05, Kolmogorov-Smirnov test, n= 30(GFP+CNO s), 42 (hM4Di+CNO s), 38(GFP+CNOp) , 19(hM4Di+CNOp) cells per group from 5 mice per group.

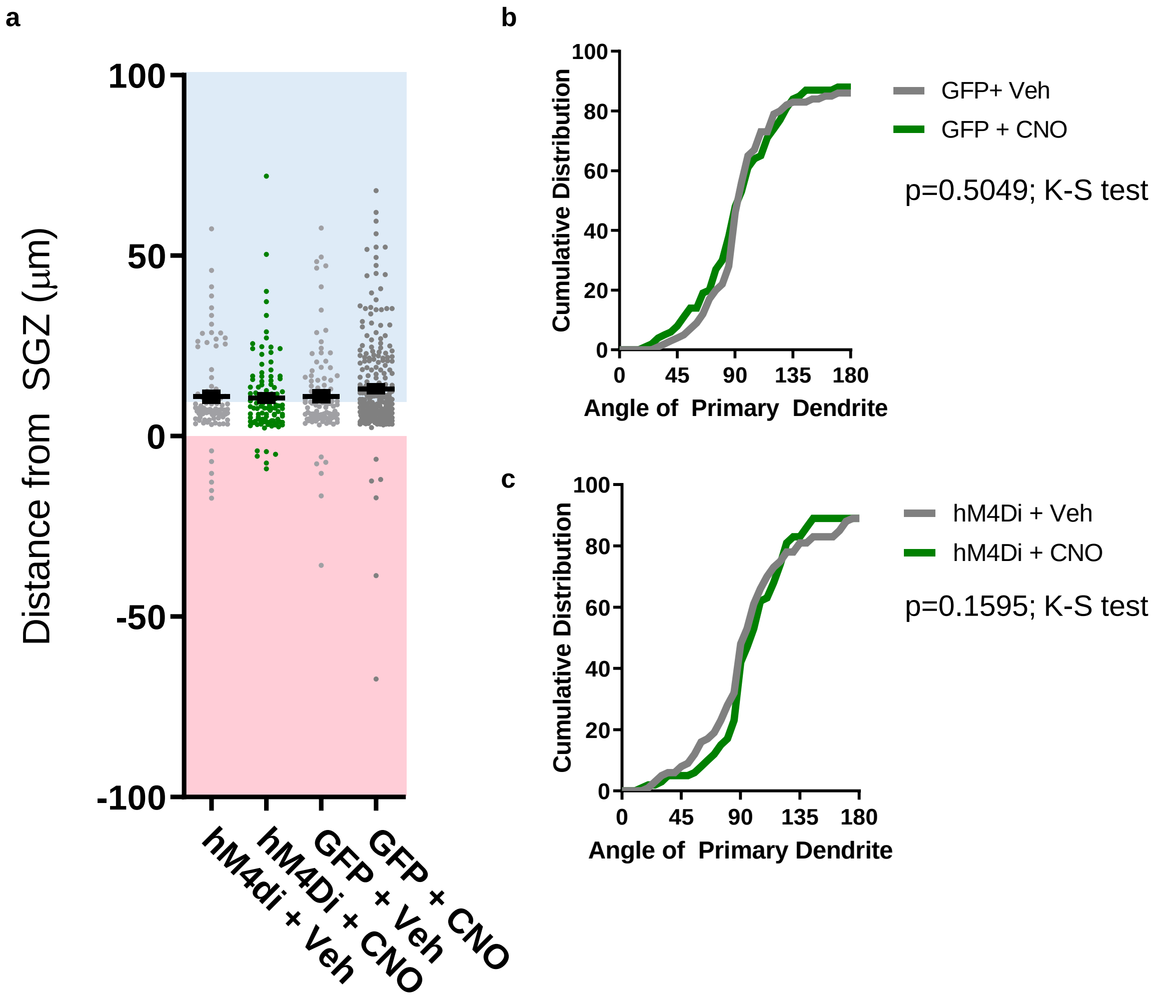

Figure S5. Silencing aberrant mature neurons does not alter migration or dendrite angle. (A) Silencing mature neurons in the pilo group does not alter migration. (B) Cumulative distribution of dendrite angle in GFP group shows no shift. (C) Cumulative distribution of dendrite angle in hM4Di group. Kolmogorov-Smirnov test. *p<0.05; n= 91, 111, 101, 253 cells per group respectively. 10 mice per group, each dot represent individual cell. All statistics calculated using two-tailed test.

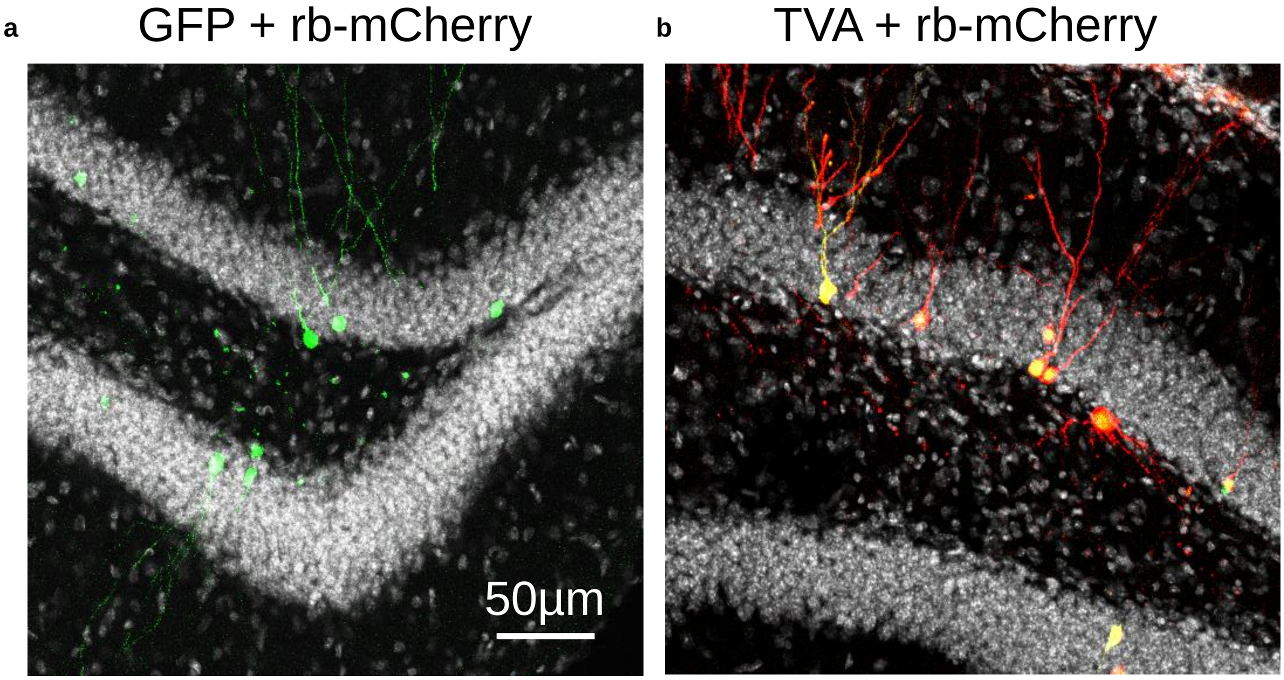

Figure S6. Pseudotyped EnvA-rabies-mCherry virus infects only TVA-gfp cells. To confirm the specificity of rb-mCherry virus, control CAG-GFP retrovirus or TVA-gfp retrovirus was injected into the hippocampus. 8 weeks later, when GFP labeled cells were fully mature, EnvA-rabies-mCherry virus was injected. 7 days later mice were perfused to confirm specificity. (A) Representative image of control GFP. No mCherry was detected confirming specificity of pseudotyped rabies virus. (B) Representative image of TVA expression with mCherry+ labeled cells.

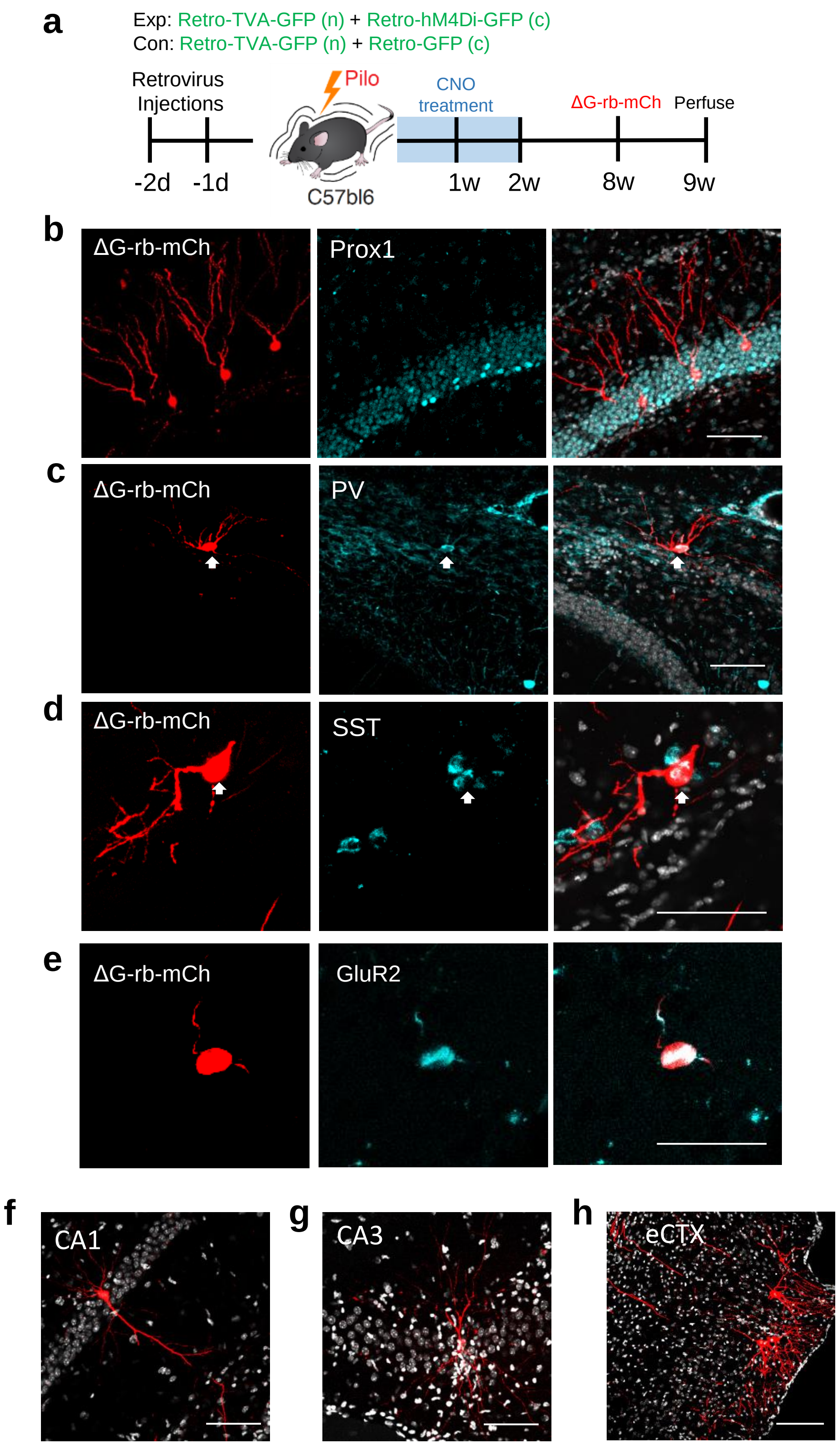

Figure S7. Input cell identification. Input cells were identified by cell specific markers. (A) Experimental design. (B) Dentate granule cells expressing Prox1. Scale bar equals 50µm. (C) Parvalbumin (PV) interneurons were identified by PV+ costaining. Scale bar equals 50µm. (D) Somatostatin (SST) interneurons, and (E) mossy cells identified with Glur2. Scale bars equal 20µm. Pyramidal cells of the (F) CA1, (G) CA3, and (H) entorhinal cortex (eCTX) were identified by morphology and spatial location. Scale bars equal 50µm.

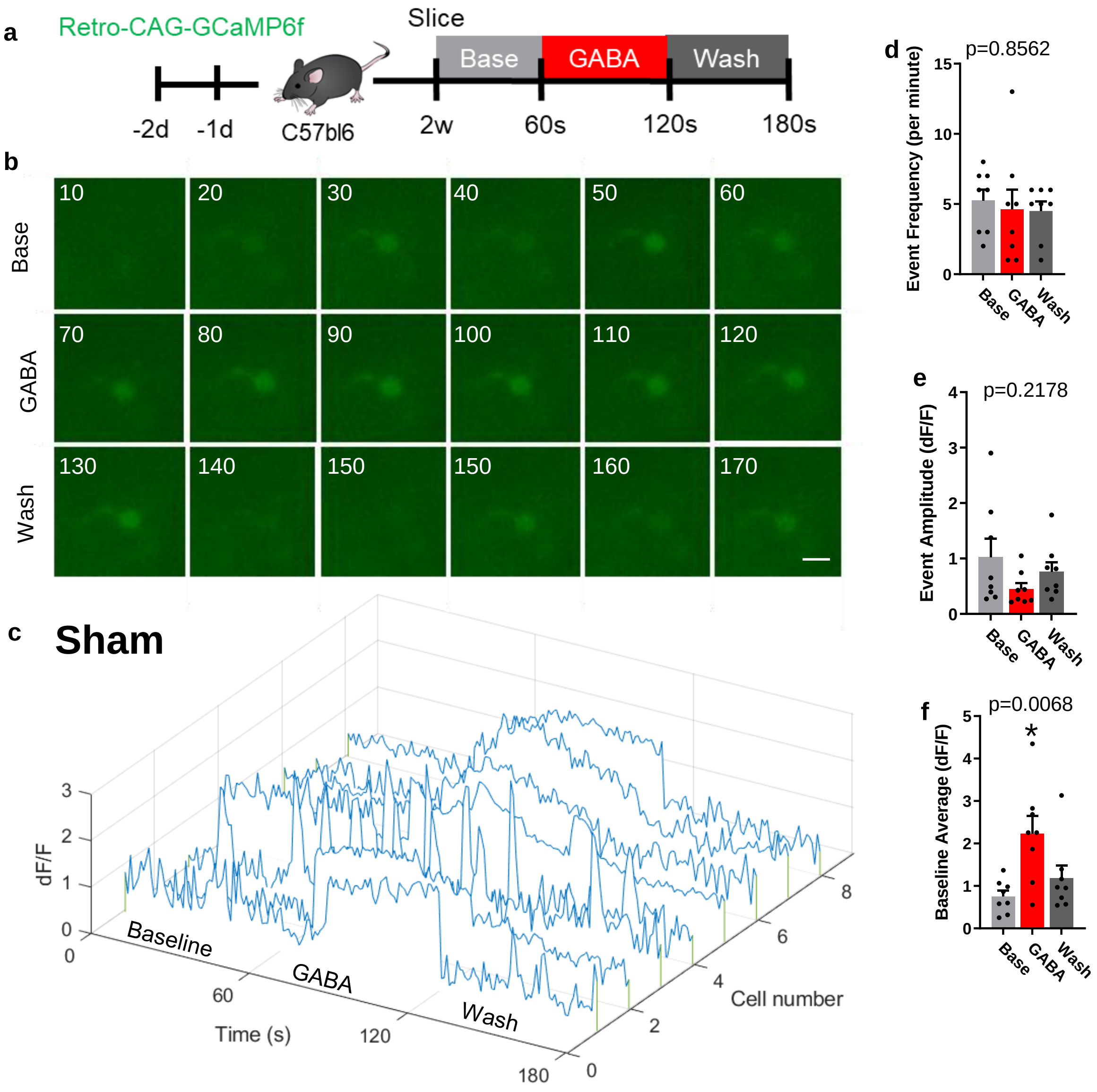

Figure S8. Calcium transients in immature adult-born GCs is GABA responsive. (A) Experimental schematic. (B) Time lapse images from immature abGCs expressing GCaMP6f in response to GABA application. (C) Representative traces from all cells in the dataset (n=8). (D) Quantification of event frequency. (E) Quantification of event amplitude. (F) Quantification of average baseline fluorescence. *p<0.05, ANOVA with repeated measures. n=8 cells per group, from 4 mice. Error bars represent SEM. All statistics calculated using two-tailed test.

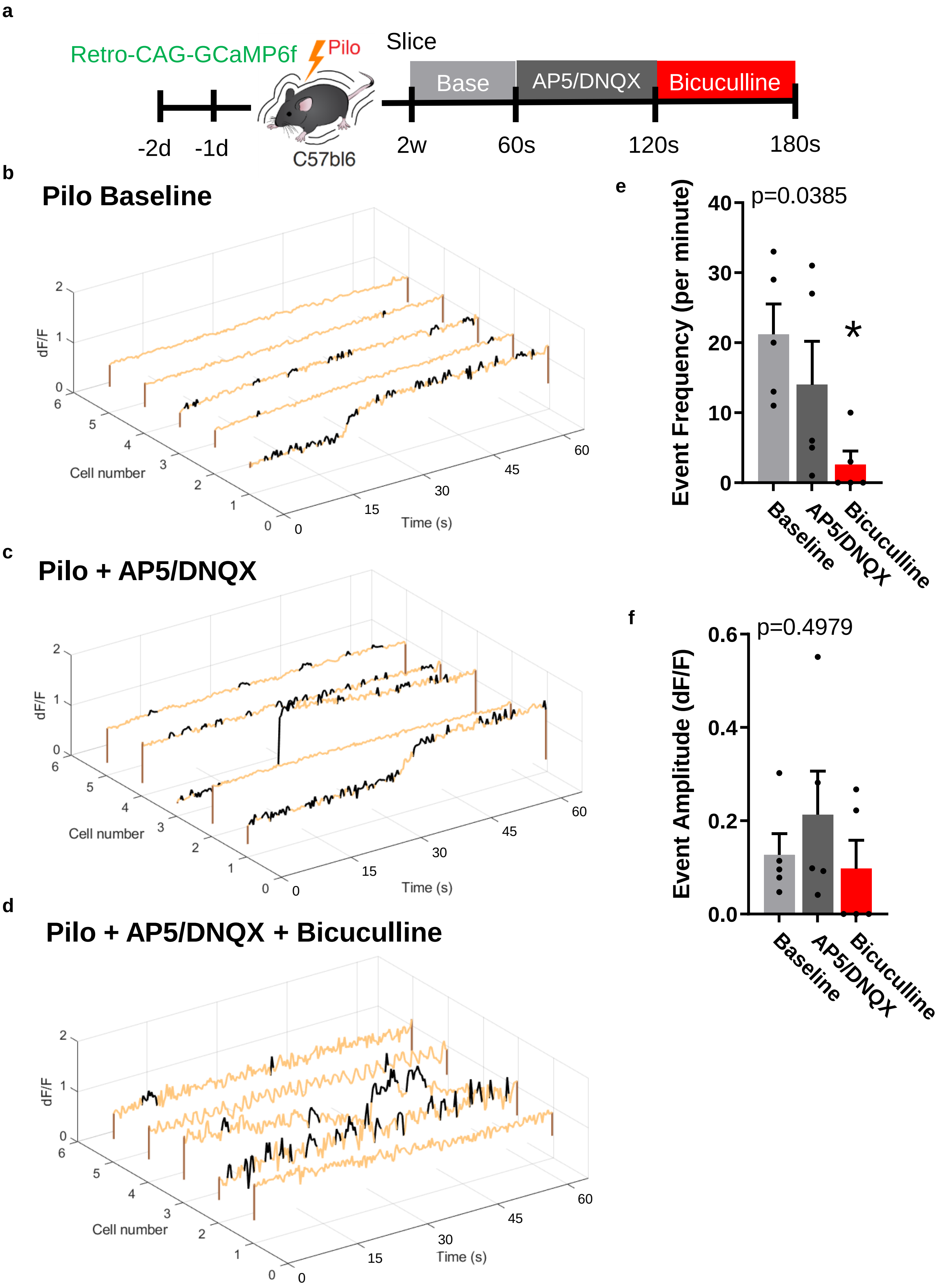

Figure S9. Calcium transients in immature aberrant adult-born GCs is not glutamate dependent. (A) Experimental schematic. (B) Representative calcium traces of all 2 week old aberrant adult-born granule cells at baseline. (C) Representative traces from bath application of glutamatergic antagonist cocktail of 10µM AP5/DNQX. (D) Representative traces from the addition of 10µM bicuculline. (E) Quantification of calcium event frequency. (F) Quantification of calcium event amplitude. Events are identified in black. *p<0.05, ANOVA with repeated measures. n=5 cells from 3 mice. Error bars represent SEM. All statistics calculated using two-tailed test.

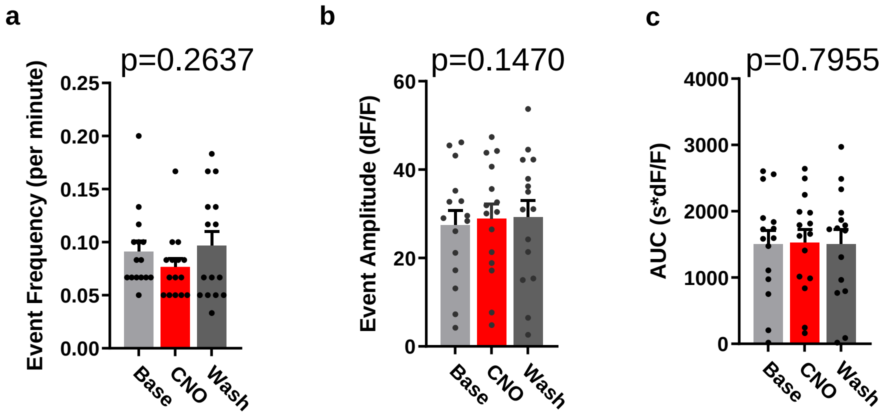

Figure S10. CNO did not alter hM4Di negative cells. (A) Quantification of event frequency. (B) Quantification of event amplitude. (C) Quantification of area under the curve (AUC). *p<0.05, ANOVA with repeated measures. n=15cells from 4 mice. Error bars represent SEM. All statistics calculated using two-tailed test.

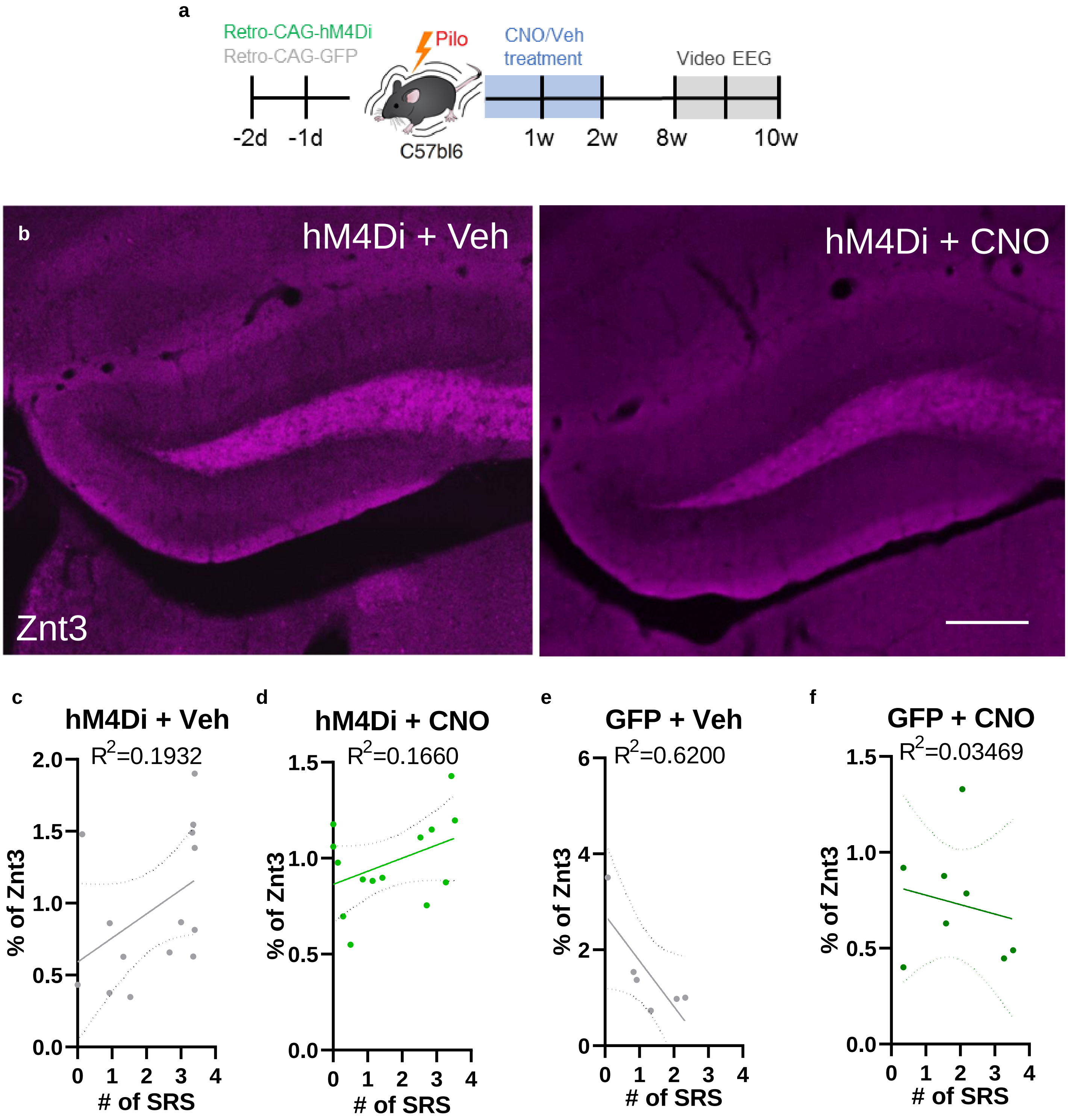

Figure S11. Mossy fiber sprouting, measured by Znt3 staining, does not change when aberrant neurogenesis is silenced. (A) Experimental design of silencing aberrant neurogenesis in pilo model of epilepsy. (B) Representative images of the dentate gyrus showing Znt3 expression. Mossy fiber sprouting after pilocarpine is seen in the hilus and the outer molecular layer. (C) There is a modest positive correlation in the number of seizures and the amount of Znt3 measured in the hM4Di + Veh group. (D) This was unchanged when CNO was administered to prevent aberrant neurogenesis. In the control GFP groups, (E) GFP + Veh and (F) GFP + CNO, there was a negative correlation in the number of SRS and Znt3 staining. Znt3 staining for mice without pilocarpine (e.g. sham mice injected with hM3Dq) was absent, even in mice with SRS (Fig. 1). Each dot represent a single animal. Scale bar equals 500µm.

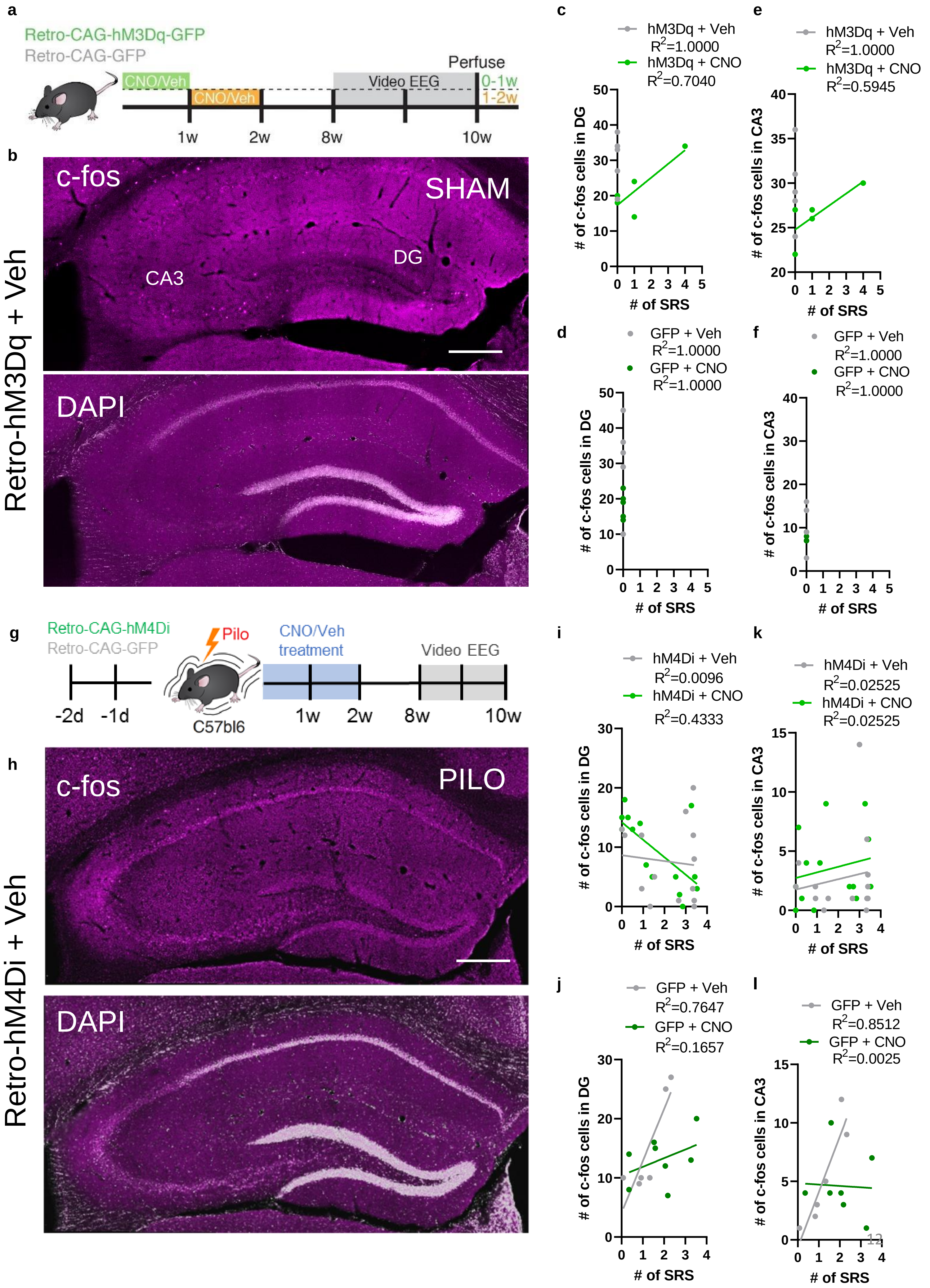
a
Retro-CAG-hM3Dq-GFP
Retro-CAG-GFP
CNO/Veh
CNO/Veh
Video EEG
Perfuse
0-1w
1-2w
1w
2w
8w
10w
b
c-fos
SHAM
CA3
DG
DAPI
Retro-hM3Dq + Veh
c
hM3Dq + Veh
R2=1.0000
hM3Dq + CNO
R2=0.7040
# of c-fos cells in DG
# of SRS
e
hM3Dq + Veh
R2=1.0000
hM3Dq + CNO
R2=0.5945
# of c-fos cells in CA3
# of SRS
d
GFP + Veh
R2=1.0000
GFP + CNO
R2=1.0000
# of c-fos cells in DG
# of SRS
f
GFP + Veh
R2=1.0000
GFP + CNO
R2=1.0000
# of c-fos cells in CA3
# of SRS
g
Retro-CAG-hM4Di
Retro-CAG-GFP
Pilo
CNO/Veh
treatment
Video EEG
-2d
-1d
C57bl6
1w
2w
8w
10w
h
c-fos
PILO
DAPI
Retro-hM4Di + Veh
i
hM4Di + Veh
R2=0.0096
hM4Di + CNO
R2=0.4333
# of c-fos cells in DG
# of SRS
k
hM4Di + Veh
R2=0.02525
hM4Di + CNO
R2=0.02525
# of c-fos cells in CA3
# of SRS
j
GFP + Veh
R2=0.7647
GFP + CNO
R2=0.1657
# of c-fos cells in DG
# of SRS
l
GFP + Veh
R2=0.8512
GFP + CNO
R2=0.0025
# of c-fos cells in CA3
# of SRS

Figure S12. Putative output of adult-born GCs, measured by c-fos, is not effected by DREADD manipulation. (A) In the sham mice that were injected with hM3Dq to activate immature GCs, c-fos activation was measured at 10 post injection. (B) Representative images of control mice with c-fos in the dentate gyrus (DG) and CA3. Sparsely labeled c-fos was observed in the DG. Scale bar equals 100 µm. Sharpness in pilo image was increased to distinguish boundary of DG and CA3. (C) Quantification of the number of c-fos labeled GCs and seizure frequency. There was an increased correlation of c-fos activation with hM3Dq activation and the number of spontaneous seizures. However, mice with no seizures had just as many c-fos activated GCs. (D) Quantification of c-fos activated cells in DG in the control GFP+Veh and GFP+CNO group. (E) Quantification of c-fos activated cells in the CA3, the primary output of the DG. There was a positive correlation with the c-fos activation and SRS frequency, however, hM3Dq activation did not change the number of c-fos activated CA3 neurons. (F) Quantification of the control GFP groups. Despite not having any seizures, there were similar number of c-fos activations. (G) Similarly with hM4Di activation to silence aberrant neurogenesis in pilo model, there was little effect on c-fos activation. (H) In pilo, c-fos expression appears elevated throughout the DG and CA3. For quantification, only cells with expression above baseline were included. Scale bar equals 500µm. Pilo image sharpness was increased to distinguish DG and CA3 boundary. (I) In the DG, there was no correlation between c-fos expression and SRS, and hM4DI+CNO did not appear to change. (J) Quantification of the GFP controls for DG expression of hM4Di+CNO did not, however CNO alone might reduce the number of c-fos activated cells. (K) Quantification of CA3 c-fos expression in the CA3. hM4Di+CNO does not change SRS. (L) GFP control for CA3. Like the DG, CNO alone appears to suppress c-fos expression in pyramidal neurons. Each dot represents an individual mouse.
