# Supplementary material for: A critical period of neuronal activity results in aberrant neurogenesis rewiring hippocampal circuitry in a mouse model of epilepsy
Source: Nat Commun. 2021 Mar 3;12:1423. doi: 10.1038/s41467-021-21649-8 (PMC7930276; doi:10.1038/s41467-021-21649-8)
Supplement: Supplementary file 12 — Reporting Summary [file 41467_2021_21649_MOESM12_ESM.pdf]

nature research

Corresponding author(s): Jenny Hsieh

Last updated by author(s): Jan 27, 2021

Reporting Summary

Nature Research wishes to improve the reproducibility of the work that we publish. This form provides structure for consistency and transparency in reporting. For further information on Nature Research policies, see our Editorial Policies and the Editorial Policy Checklist.

Statistics

For all statistical analyses, confirm that the following items are present in the figure legend, table legend, main text, or Methods section.

n/a	Confirmed	
☐	☒	The exact sample size (*n*) for each experimental group/condition, given as a discrete number and unit of measurement
☐	☒	A statement on whether measurements were taken from distinct samples or whether the same sample was measured repeatedly
☐	☒	The statistical test(s) used AND whether they are one- or two-sided<br>*Only common tests should be described solely by name; describe more complex techniques in the Methods section.*
☐	☒	A description of all covariates tested
☐	☒	A description of any assumptions or corrections, such as tests of normality and adjustment for multiple comparisons
☐	☒	A full description of the statistical parameters including central tendency (e.g. means) or other basic estimates (e.g. regression coefficient) AND variation (e.g. standard deviation) or associated estimates of uncertainty (e.g. confidence intervals)
☐	☒	For null hypothesis testing, the test statistic (e.g. *F*, *t*, *r*) with confidence intervals, effect sizes, degrees of freedom and *P* value noted<br>*Give P values as exact values whenever suitable.*
☒	☐	For Bayesian analysis, information on the choice of priors and Markov chain Monte Carlo settings
☒	☐	For hierarchical and complex designs, identification of the appropriate level for tests and full reporting of outcomes
☐	☒	Estimates of effect sizes (e.g. Cohen's *d*, Pearson's *r*), indicating how they were calculated

Our web collection on statistics for biologists contains articles on many of the points above.

Software and code

Policy information about availability of computer code

Data collection	All software used for data collection is commercially available. For mouse EEG studies, data was collected using Penomah software from Data Sciences International version 3.0. For all imaging studies, Leica Acquisition Suite Xv3.4.2.18368 was used to collect calcium imaging data and confocal imaging data.
Data analysis	Commercially available software was used for data analysis. For mouse EEG, Neuroscore (Data Sciences International v3.0) and Leica Acquisition Suite X v3.4.2.18368 was used to analyze calcium imaging data. We additionally used the publically available PeakCaller MATLAB script from Hussman Institute for Autism for identifying calcium events Standard MATLAB functions were also used for analyzing calcium imaging with MATLAB vR2017b.<br><br>For dendrite analysis and morphology, Neurolucida SOftware v10 from MBF Biosciences, MicroBrightField, Inc. and Image J software v1.52C (NIH).<br><br>For statistics Graphpad Prism v8.4.3 was used.

For manuscripts utilizing custom algorithms or software that are central to the research but not yet described in published literature, software must be made available to editors and reviewers. We strongly encourage code deposition in a community repository (e.g. GitHub). See the Nature Research guidelines for submitting code & software for further information.

Data

Policy information about availability of data

All manuscripts must include a data availability statement. This statement should provide the following information, where applicable:

- Accession codes, unique identifiers, or web links for publicly available datasets
- A list of figures that have associated raw data
- A description of any restrictions on data availability

All data supporting the findings of this study are provided within the paper and its supplementary information. A source data file is provided with this paper. All additional information will be made available upon reasonable request to the authors.

Field-specific reporting

Please select the one below that is the best fit for your research. If you are not sure, read the appropriate sections before making your selection.

☒ Life sciences ☐ Behavioural & social sciences ☐ Ecological, evolutionary & environmental sciences

For a reference copy of the document with all sections, see nature.com/documents/nr-reporting-summary-flat.pdf

Life sciences study design

All studies must disclose on these points even when the disclosure is negative.

Sample size	Sample sizes for all data sets were determined using power analysis based on pilot data or previously published data applicable to the specific experiment. Cho et al., 2015 (https://doi.org/10.1038/ncomms7606), Vivar et al., 2015 (doi: 10.1038/ncomms2101), and Ge et al. 2006 (doi: 10.1038/nature04404.) were used to determine sample sizes for Figures 1-4, Supplementary Figures 1-7 and 11-12. Heigele et al. 2016 (doi:10.1038/nn.4218) was used to determine sample sizes for Figure 5-6 and Supplementary figures 8-10.
Data exclusions	Data was excluded based on predetermined criteria described below:<br>For all experiments involving virus injections, mice were excluded from the study if no labeled cells were present.<br>For pilocarpine model of epilepsy, mice that did not reach status epilepticus within 1 hour of pilocarpine injection and sustained status epilepticus for 3 hours were excluded from the study.<br>For calcium imaging, cells from mice that received pilocarpine were excluded if they showed no response to bicuculline suggesting the cells were dead.
Replication	Each data set is a combination of 2-3 successful experimental replications. All data was included in the final analysis unless excluded as described above. All attempts were successful.
Randomization	Mice used in this study were wildtype C57bl6 and housed with 5 mice per cage. Each cage was assigned at random to an experimental group.
Blinding	Data analysis was performed by a blinded researcher to the experimental groups. For all experiments, the individual that performed the experiments was independent of the individual that analyzed the data.

Reporting for specific materials, systems and methods

We require information from authors about some types of materials, experimental systems and methods used in many studies. Here, indicate whether each material, system or method listed is relevant to your study. If you are not sure if a list item applies to your research, read the appropriate section before selecting a response.

Materials & experimental systems

n/a	Involved in the study	
☐	☒	Antibodies
☐	☒	Eukaryotic cell lines
☒	☐	Palaeontology and archaeology
☐	☒	Animals and other organisms
☒	☐	Human research participants
☒	☐	Clinical data
☒	☐	Dual use research of concern

Methods

n/a	Involved in the study	
☒	☐	ChIP-seq
☒	☐	Flow cytometry
☒	☐	MRI-based neuroimaging

Antibodies

Antibodies used	Primary antibodies used: chicken anti-GFP (1:500; Aves Labs GFP-1010), rabbit-anti-Prox1 (1:500, Millipore AB5475), rat anti-somatostatin (SST clone YC7; 1:500, Millipore MAB 354), mouse anti-parvalbumin (PV; 1:500, Millipore MAB1572), rabbit anti-GluR2

	(1:500; Millipore Sigma AB1768-I), mouse anti-c-Fos (1:500; Santa Cruz sc-8047; clone D-1), rabbit anti-ZNT3 (1:500; Millipore Sigma ABN994). For double labeling, primary antibodies were separately with GFP amplification first (e.g. GFP*/Proxl, GFP*/PV, GFP*/SST). Secondary antibodies used: biotin anti-chicken (1:200; Jackson ImmunoResearch 703-065-155), CY5 secondary against rabbit (1:200; Jackson ImmunoResearch 711-005-152), rat (1:200; Jackson ImmunoResearch 712-005-153), and mouse (1:200; Jackson ImmunoResearch 715-005-151).
Validation	For all primary antibodies, we validated using immunohistochemistry in mouse brain tissue and provided representative images that can be found in text. All antibodies are commercially available and described below how they were validated using immunohistochemistry (IH), immunocytochemistry (fC) or western blot (WB) by the manufacturer.<br>Chicken anti-GFP was validated by manufacturer with WB amd IHC using transgenic mice expressing GFP gene product.<br>Rabbit anti-Proxl was validated by manufacturer using IH and IC in rat, human, zebrafish, and mouse.<br>Rat anti-somatostatin was validated by manufacturer using IH and IC in human, rat, and rabbit.<br>Mouse anti-parvalbumin was validated by manufacturer using IH, IC, and WB in human, mouse, rat, and rabbit.<br>Rabbit anti-GluR2 was validated using IH and WB in mouse, rat, and human.<br>Mouse anti c-fos was validated using IH, IC, and WB in mouse, rat, and human.<br>Rabbit anti-znt3 was validated using WB and IHC in human, mouse, and monkey.

Eukaryotic cell lines

Policy information about cell lines

Cell line source(s)	293T cells used for packaging virus are deposited in the Hsieh lab.
Authentication	293T cells used for packaging virus were not authenticated
Mycoplasma contamination	293T cells used for packaging virus were not tested for mycoplasma contamination
Commonly misidentified lines (See ICLAC register)	no commonly misidentified cell lines were used in this study.

Animals and other organisms

Policy information about studies involving animals; ARRIVE guidelines recommended for reporting animal research

Laboratory animals	All mice (C57bl/6, all female) were purchased from Envigo at 5-6 weeks of age and housed in the animal facility with twelve-hour light/dark cycle, room temperature 65-75F(18-23C) with 40-60% humidity. Mice were given access to standard chow and water ad libitum. All mice were group housed (5 per cage) except during video-EEG monitoring where they were single housed.
Wild animals	This study did not use wild animals
Field-collected samples	This study did not use field-collected samples
Ethics oversight	All experiments were performed in compliance with the animal care guidelines issued by the National Institutes of Health and by the Institutional Animal Use and Care Committee at The University of Texas at San Antonio (Protocol #MU112).

Note that full information on the approval of the study protocol must also be provided in the manuscript.
